# Association Between Baseline and Changes in Early Neutrophil-to-Lymphocyte Ratio on Survival in Patients with Metastatic Bladder Carcinoma Treated with Immunotherapy

**DOI:** 10.3390/medicina60122103

**Published:** 2024-12-22

**Authors:** Ezgi Değerli, Çağatay Arslan, Fatih Selçukbiricik, Ömer Fatih Ölmez, Dilek Erdem, Jamshid Hamdard, Mesut Yılmaz, Rumeysa Çolak, Caner Kapar, Mustafa Erman, Fatih Kuş, Deniz Tural

**Affiliations:** 1 Department of Medical Oncology, Bakırköy Dr. Sadi Konuk Training and Research Hospital, 34147 Istanbul, Turkey; mesutyilmaz12@yahoo.com (M.Y.); colak.rmys@gmail.com (R.Ç.); kaparcaner@gmail.com (C.K.); 2 Department of Medical Oncology, Medical Park İzmir Hospital, 35230 Izmir, Turkey; arslancagatay@yahoo.com; 3 Department of Medical Oncology, Faculty of Medicine, Koc University, 34450 Istanbul, Turkey; fsbiricik@yahoo.com (F.S.); deniztural@gmail.com (D.T.); 4 Department of Medical Oncology, Medipol University Hospital, 34810 Istanbul, Turkey; olmezof@gmail.com (Ö.F.Ö.); jamshidhamdard@hotmail.com (J.H.); 5 Department of Medical Oncology, Medical Park Samsun Hospital, 55200 Samsun, Turkey; dilekgurgenyatagi@yahoo.com; 6 Department of Medical Oncology, Faculty of Medicine, Hacettepe University, 06100 Ankara, Turkey; ermanm1968@gmail.com (M.E.); fatihkush@hotmail.com (F.K.)

**Keywords:** immunotherapy, baseline NLR, bladder cancer, change in NLR, overall survival

## Abstract

*Background and Objectives*: A high baseline neutrophil-to-lymphocyte ratio (NLR) is a poor prognostic factor in various cancers. However, its predictive role in metastatic bladder cancer (mBC) treated with immunotherapy is unclear. In this study, we aimed to investigate the relationship between the baseline and change in NLR and overall survival in mBC patients treated with immunotherapy, with the potential to significantly impact patient care. *Materials and Methods*: A retrospective analysis was conducted on 56 mBC patients who received second-line immunotherapy after progressing on platinum-based chemotherapy. Patients were classified into high and low NLR groups using a cutoff value of 3.3. A further division was made based on NLR changes after two cycles of immunotherapy: whether NLR increased (≥10%) or decreased (≥10%). The endpoint was to estimate the association between clinicopathological features and survival outcomes. *Results*: The study included 56 patients, with a median age of 66.6 years and a male-to-female ratio of 2.3:1. A low baseline NLR was associated with better OS than a high baseline NLR (*p* = 0.005). After two immunotherapy cycles, patients with a decreased NLR (≥10%) had significantly longer OS than those with an increased NLR (≥10%), regardless of the baseline NLR (*p* = 0.003). The overall median survival was 15 months, with 10 months for the NLR-increased group and not reached for the NLR-decreased group. *Conclusions*: Our study highlights the potential of baseline NLR and early changes in NLR as valuable prognostic markers for mBC patients receiving immunotherapy. Elevated neutrophils and lymphopenia negatively impact prognosis and treatment effectiveness, and NLR shows promise as a prognostic marker, inspiring further research and potential improvements in patient care.

## 1. Introduction

Bladder cancer (BC) is the tenth most commonly diagnosed cancer worldwide, with approximately 550,000 new cases annually [1,2]. Urothelial carcinoma is the dominant histologic type and presents a significant cause of mortality, with a 5-year survival rate of about 5% for the metastatic disease [3]. Approximately 10–15% of patients are diagnosed with metastatic bladder cancer (mBC) at the time of presentation. As such, developing new treatment strategies is crucial for metastatic bladder cancer patients with a poor prognosis [4]. The combination of pembrolizumab and enfortumab vedotin is the preferred regimen of eligible patients for cisplatin treatment [5]. However, for patients who cannot receive this combination treatment, the preferred path is platinum-based chemotherapy. Single-agent immunotherapy in Programmed Death Ligand 1 (PD-L1)-positive patients is recommended in platin-ineligible patients. For second-line therapy in metastatic disease, immunotherapy is recommended regardless of PD-L1 positivity [6]. Although PD-L1 positivity predicts a benefit from immunotherapy in first-line treatment, it is not clear under what conditions patients will benefit from immunotherapy as a second-line treatment. Therefore, predictive markers must be detected in peripheral blood to show that immunotherapy is beneficial for patients with metastatic bladder cancer. Complete blood count tests can easily determine the morphology of blood and can be routinely checked before each treatment. In many malignancies, the neutrophil-to-lymphocyte ratio (NLR) is strongly associated with systemic inflammation and retains a substantial prognostic value [7,8]. In retrospective studies and immunotherapy for bladder cancer, a high baseline NLR is associated with a poor prognosis and is an independent prognostic factor [9,10].

In the literature, studies have confirmed that a high baseline NLR and an early increase in NLR after immunotherapy can predict the negative effect of treatments for metastatic renal cell carcinoma, malign melanoma, gastrointestinal cancer, and non-small cell lung cancer [11,12,13,14,15]. Studies have shown that NLR is a characteristic predictor of survival in bladder cancer patients [8,16]. However, there are no precise data that elucidate that an early change in NLR during treatment is related to the prognosis of patients with mBC who have received immunotherapy. In this study, we aimed to investigate the association between baseline NLR and changes in NLR after treatment and the outcomes of mBC patients treated with immunotherapy.

## 2. Methods

### 2.1. Patients

We conducted a retrospective analysis of all patients with metastatic breast cancer (mBC) who were treated with immunotherapy. All patients were receiving second-line treatment after progressing with platinum-based chemotherapy in the first line. Data were collected from patients’ medical records and the hospital database, including parameters such as demographic information, ECOG performance status, smoking status, baseline neutrophil-to-lymphocyte ratio (NLR) at the start of immunotherapy, NLR at 6 (±2) weeks after the initiation of treatment, duration of treatment, best response to immunotherapy, and overall survival. Overall survival (OS) was defined as the time from the start of immunotherapy until death. This study was approved by the local ethics committee (Protocol No: 2023/488).

The NLR cutoff value was determined via receiver operating characteristic (ROC) analysis. ROC analysis yielded an area under the curve (AUC) of 0.711, 95% CI: 0.564–0.859, *p*: 0.007, with a sensitivity of 70.4% and specificity of 72.4%. Patients were initially divided into two groups: high NLR and low NLR. The change in NLR at 6 weeks was calculated using the formula {[(NLR at week 6/NLR at baseline) − 1] × 100}. Based on the average value, patients were further classified into two groups: the NLR-increased group (≥10% increase) and the NLR-decreased group (≥10% decrease). Survival analysis was performed to compare the outcomes between the NLR-elevated and -decreased groups, regardless of the baseline NLR.

### 2.2. Statistical Analysis

Statistical analysis utilizing IBM SPSS Statistics for Windows was performed using version 25.0 (Statistical Package for the Social Sciences, IBM Corp., Armonk, NY, USA). The descriptive statistics are presented as the number and percentage for categorical variables and the median (min–max) for continuous variables. The data were examined with Kolmogorov–Smirnov values regarding the normality assumptions (*p* < 0.05). Therefore, a Whitney U test was used for two-group comparisons, and ROC analysis was used to predict mortality using the NLR value. Regarding the ROC analysis, increased and decreased NLR groups were determined according to the percentage change in NLR over 6 weeks. Pearson chi-square and Fisher’s exact tests were used to compare the categorical variables. The Kaplan–Meier method was utilized to compare the survival times between the various clinical parameter groups, with a *p* < 0.05 value considered to be statistically significant.

## 3. Results

### Patient Characteristics and Outcomes

**Figure 1:**
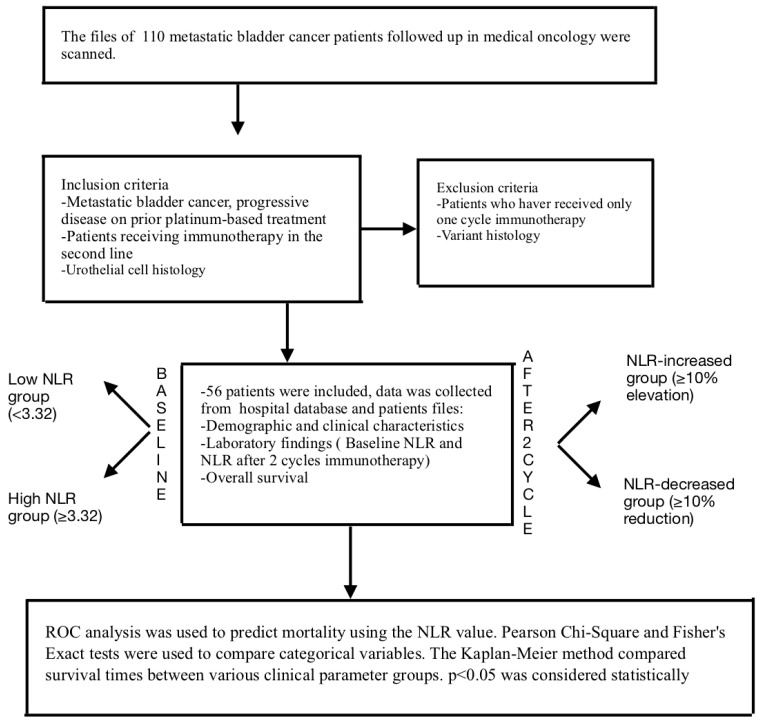
Flowchart of the materials and methods.

This comprehensive study included a total of 56 metastatic bladder cancer patients who had progressed under platinum-based chemotherapy and had received second-line immunotherapy. All patients received identical immunotherapy treatments (Figure 1). The median age at the start of immunotherapy was 66.6 years (39–85). All patients had a histology of urothelial carcinoma. The gender distribution of the patients was a male-to-female ratio of 2.3:1. Most patients (91.1%) had ECOG PS 1 or greater, and 50 (89.3%) had a history of smoking. Among the included patients, 33 (58.8%) had lymph node metastasis, 10 (17.9%) had lung metastasis, 26 (46.4%) had bone metastasis, and 17 (30.4%) had liver metastasis at baseline. Regardless of NLR, no significant difference was found between the metastasis sites and survival (for bone metastasis *p* = 0.885, for lung metastasis *p* = 0.419, for lymph node metastasis *p* = 0.196, and for liver metastasis *p* = 0.174).

**Figure 2:**
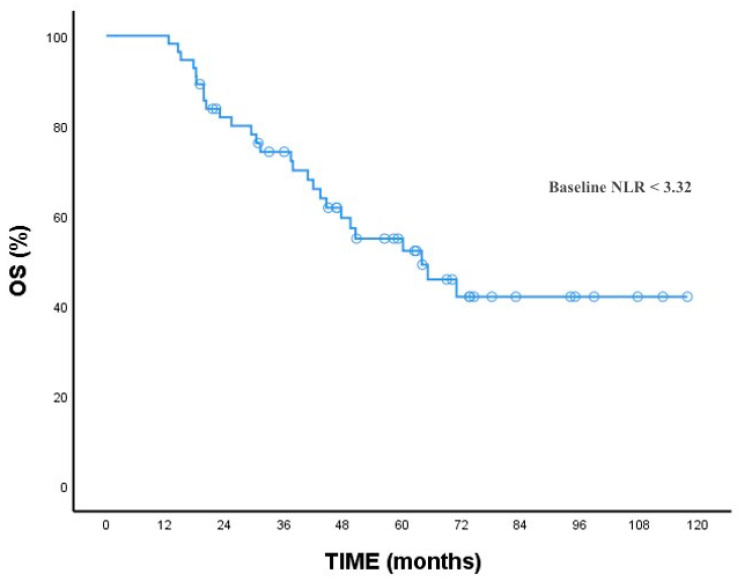
OS for baseline NLR.

At baseline, the NLR mean (range) level was 3.5 (1.50–18.52). The optimal cutoff values of NLR were determined via the ROC analysis (cutoff: 3.3). Based on these values, patients were divided into two groups (baseline low NLR group and baseline high NLR group). The patients’ clinicopathological characteristics, treatment response, and survival analysis were compared with the baseline NLR high and low groups. The comparative analysis of all patients and the two groups is summarized in Table 1. In summary, baseline NLR was significantly higher in patients with lymph node metastasis and liver metastasis at diagnosis than those without metastasis (*p*: 0.001, *p*: 0.015, respectively). As expected, survival was better in patients with a lower baseline NLR (Figure 2). Overall survival was significantly higher in the baseline low NLR group (*p*: 0.005).

The mean (range) level of NLR after two courses of immunotherapy was 2.7 (0.86–25.17). After two cycles of immunotherapy, the NLR level of patients showed a change of at least 10 percent, according to the ROC analysis. The patients were divided into two groups: those with a 10% decrease in NLR level and those with a 10% increase based on the alteration of the inflammatory biomarkers after two cycles of immunotherapy (NLR-increased group and NLR-decreased group). The clinical features of these two groups are summarized in Table 2.

**Figure 3:**
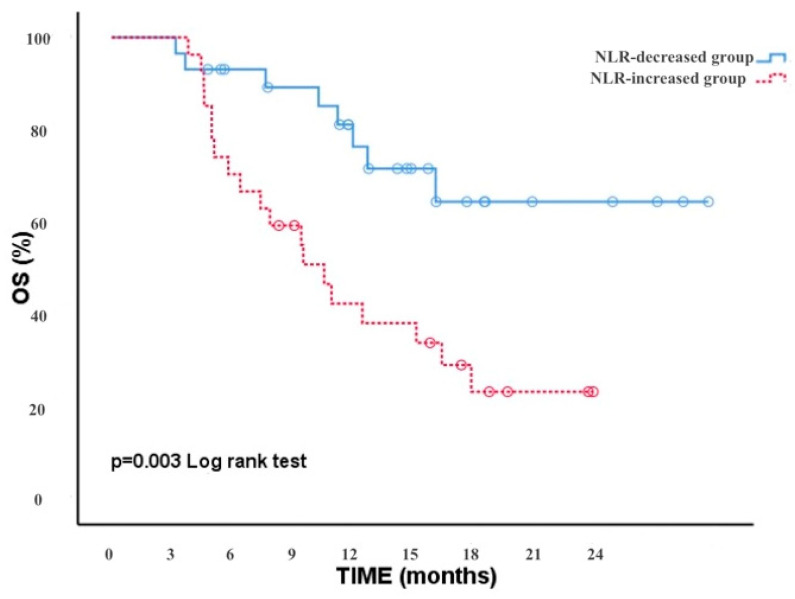
OS in the NLR decreasing and increasing groups.

Moreover, we analyzed the correlation between the NLR-increased/decreased groups and OS. The results showed that patients with a decreased NLR after two treatment cycles had a significantly longer OS than patients with an increased NLR [*p* = 0.003, (95% CI, 7.95–12.75)]. At the time of the analysis, the OS of patients treated with immunotherapy was 15 months (95% 10.20–21.75). During a follow-up period of approximately 24 months, the median OS was 10 months in the NLR-increased group and less in the NLR-decreased group (Figure 3).

## 4. Discussion

Bladder cancer, the tenth most common cancer type worldwide, is a significant health concern. In 2023, more than 550,000 people were diagnosed with bladder cancer globally, and, tragically, over 220,000 people succumbed to the metastatic form of the disease [1]. In recent years, the expected survival rate for bladder cancer, particularly in cases where immune sensitivity is known, has improved to up to 32 months with combination studies involving immunotherapy [5]. However, for patients who cannot access combination therapy, such as those who are platinum-resistant or platinum-ineligible, single-agent immunotherapy is the only option in sequential treatment. The effect of inflammatory markers on survival has been demonstrated in patients treated with immunotherapy. The association between high NLR levels and a poor prognosis has been recognized in many tumors [17]. A high NLR at all stages of bladder cancer has been associated with poor survival [18,19]. However, the fact that inflammatory markers may change with immunotherapy may also affect the prognosis. We determined for mBC patients treated with immunotherapy that an early decline (decrease ≥ 10%) 6-week NLR was associated with a longer OS than an increased 6-week NLR, regardless of the baseline NLR levels. Our data suggest that baseline NLR and early-change NLR appear to be easily obtainable prognostic markers for metastatic bladder cancer patients treated with immunotherapy.

Immunotherapy targets both the tumor and tumor microenvironment [20], which produces immune cells, including neutrophils. Elevated neutrophil levels can cause the synthesis of proinflammatory cytokines, chemokines, vascular endothelial growth factors, tumor angiogenesis, and tumor progression [21,22]. In addition, leukocytes constitute more than half of the total tumor tissue and play a critical role in cytotoxic cell death [23]. In other words, a high neutrophil–lymphocyte ratio creates a predisposition for tumor progression. Lymphopenia causes the immune response to be low in metastatic advanced-stage cancer patients, and therefore, the effectiveness of immunotherapy is insufficient [24]. It has been shown in the literature that an increase in neutrophils and a decrease in lymphocytes during treatment with immunotherapy have a negative impact on the prognosis of cancer patients [25].

Bladder cancer, known for its immunogenic nature, has been the focus of significant research. Intravesical Bacillus Calmette–Guerin (BCG) treatment has been shown to stimulate an innate immune response locally and systemically for stage I bladder cancer [26,27]. The effectiveness of pembrolizumab in in situ bladder cancer has been proven [28]. Our research, which suggests that a 10 percent change in NLR at six weeks can affect survival, could potentially influence future treatments. While data on the effects of changes in NLR in metastatic bladder cancer are limited, a similar study identified a baseline cutoff of 3% for NLR and found that a change of 15% was prognostically significant [29]. These figures align closely with our findings, and the slight differences can be attributed to variations in treatment modalities and the number of patients involved. In other malignancies, this rate was found to be around 25–30%. Bartlett et al. showed that an elevated baseline NLR (≥5%) and an increased NLR (≥30%) during early treatment are prognostic for OS in patients who have melanomas treated with immunotherapy [13]. Lalani et al. declared that an early decline (≥25%) in NLR at 6 weeks is associated with significantly improved outcomes in metastatic renal cell carcinoma patients treated with immunotherapy [11]. In Ouyang et al.’s study, a low baseline NLR (≥3.5) and an early NLR presentation are significantly associated with a better prognosis in metastatic colorectal carcinoma patients treated with immunotherapy. The subgroup analysis showed that the best prognosis belonged to the patient group with a low NLR at baseline, which decreased more with immunotherapy [15]. In Lim et al.’s data, early changes in NLR were shown to have prognostic value in metastatic non-small cell lung cancer patients treated with immunotherapy [30]. In addition, Gou et al. conducted the most comprehensive meta-analysis in the literature. Thirty-four studies were analyzed, and data from 4154 patients were presented. In their study, patients with a high baseline NLR whose NLR level increased with immunotherapy were associated with worse clinical outcomes. It has been shown that patients whose NLR levels decrease with immunotherapy have a better prognosis [31]. In this meta-analysis, only one study examines the relationship between immunotherapy and changes in NLR with the prognosis of bladder cancer. Unlike our study, Tomioka-Inagawa et al. evaluated the response of mBC patients given pembrolizumab after two cycles of immunotherapy [32]. They divided the patients into two groups: responders and non-responders. While there was no difference in the baseline NLRs between the two groups, they observed that the NLR in the patient group with a treatment response after two cycles of immunotherapy was significantly lower than in the non-responsive group. Furthermore, they showed a significant association between the NLR after two courses of immunotherapy and OS.

All patients included in our study had a histology of pure urothelial carcinoma. This type of cancer is generally believed to respond better to immunotherapy treatments. The incidence of patients with a variant histology in similar studies is around 1 percent. We believe no patients with a variant histology were present in our study due to the overall number of participants.

In a review by Claps et al., it was emphasized that a variant histology in bladder cancer exhibits different behavior patterns compared to urothelial carcinoma, and therefore, treatment modalities should also differ [33]. The authors noted that not all subtypes of variant histology should be treated the same way and that specific considerations should be made for each subtype. They highlighted that contrary to historical belief, emerging evidence is beginning to support the distinct responses of variant histologies in immunotherapy studies. The PURE-01 study demonstrated that only the lymphoepithelioma-like and squamous variants were sensitive to neoadjuvant pembrolizumab immunotherapy [34]. Additionally, the results of the phase 2 adjuvant study of durvalumab in variant histology bladder cancer are eagerly anticipated [35].

To our knowledge, this is the second similar study published in the literature. In the first study, Dionese et al. highlighted the relationship between initial NLR and changes in NLR with immunotherapy in mBC patients and prognosis. Unlike our study, an additional hematological parameter was examined, and patients with a low initial monocyte–lymphocyte ratio (MLR) had a better prognosis. Dionese et al. also emphasized that only the change in neutrophil–lymphocyte ratio with immunotherapy significantly contributes to prognosis [29]. In our study, we could not examine the MLR due to a lack of data. However, our study is the first Turkish study showing that patients with decreased NLR levels after immunotherapy in mBC have a better prognosis. Due to the extended immune response, patients with mBC who exhibited a reduced NLR following immunotherapy may experience a possible beneficial effect from two cycles of immunotherapy.

While our study provides valuable insights, it has several limitations. It is retrospective in nature and includes a limited number of patients. The short follow-up period might also impact the significance of our outcomes. Moreover, we could not collect data for concomitant medications that may have affected leukocyte count. We also could not collect data on the immune side effects that develop after immunotherapy, which may affect the immune system. While these deficiencies did not significantly affect our treatment outcomes, they will provide informative data for future combination immunotherapy regimens for mBC.

## 5. Conclusions

In conclusion, our study emphasizes that metastatic bladder cancer patients with a baseline NLR < 3.3 and those with a decrease in NLR of more than 10% after two cycles of immunotherapy have a better prognosis. Changes in NLR are an early predictive surrogate and may be an important indicator for changes in the early period of treatment. However, long-term prospective studies involving large patient populations with extended follow-up periods are needed to fully understand the potential of NLR as a prognostic marker. These studies will help determine whether a low-cost, easily accessible prognostic marker can identify those mBC patients who require long-term immunotherapy.

## Figures and Tables

**Table 1:** Baseline characteristics and comparative analysis of all patients (n = 56).

	Total N = 56	Low (<3.32) N = 29	High (≥3.32) N = 27	*p*
Age median (min–max)	66.5 (39–85)	68.0 (50–85)	66.0 (39–84)	0.617 ^c^
Sex				
Female	17 (30.4)	10 (34.5)	7 (25.9)	0.487 ^a^
Male	39 (69.6)	19 (65.5)	20 (74.1)	
Smoker status				
Active or former smoker	6 (10.7)	2 (6.9)	4 (14.8)	0.414 ^b^
Never smoked	50 (89.3)	27 (93.1)	23 (85.2)	
ECOG				
0	9 (16.1)	6 (20.7)	3 (11.1)	0.071 ^b^
1	42 (75.0)	23 (79.3)	19 (70.4)	
2	4 (7.1)	0 (0)	4 (14.8)	
3	1 (1.8)	0 (0)	1 (3.7)	
Lymph node-only metastasis				
No	23 (41.1)	18 (62.1)	5 (18.5)	0.001 ^a^
Yes	33 (58.8)	11 (37.9)	22 (81.5)	
Lung metastasis				
No	46 (82.1)	23 (79.3)	23 (85.2)	0.731 ^b^
Yes	10 (17.9)	6 (20.7)	4 (14.8)	
Bone metastasis				
No	30 (53.6)	15 (51.7)	15 (55.6)	0.774 ^a^
Yes	26 (46.4)	14 (48.3)	12 (44.4)	
Liver metastasis				
No	39 (69.6)	16 (55.2)	23 (85.2)	0.015 ^a^
Yes	17 (30.4)	13 (44.8)	4 (14.8)	

^a^: Pearson Chi Square test, ^b^: Fisher’s Exact test, ^c^: Mann Whitney U test, *p* < 0.05.

**Table 2:** Clinical characteristics of patients with a 10% increase and decrease in NLR.

	NLR		
	NLR Increased (≥10%), n = 35	NLR Decreased (≥10%), n = 21	*p*
Age median (min–max)	67.0 (39–80)	65.0 (50–85)	0.839 ^c^
Sex			
Female	13 (37.1)	4 (19)	0.154 ^a^
Male	22 (62.9)	17 (81)	
Smoker status			
Never smoked	4 (11.4)	2 (9.5)	0.599 ^b^
Active or former smoker	31 (88.6)	19 (90.5)	
ECOG			
0	5 (14.3)	4 (19)	0.812 ^b^
1	27 (77.1)	15 (71.4)	
2	2 (5.7)	2 (9.5)	
3	1 (2.9)	0 (0)	
Lymph node-only metastasis			
No	16 (45.7)	7 (33.3)	0.362 ^a^
Yes	19 (54.3)	14 (66.7)	
Lung metastasis			
No	31 (88.6)	15 (71.4)	0.152 ^b^
Yes	4 (11.4)	6 (28.6)	
Bone metastasis			
No	19 (54.3)	11 (52.4)	0.890 ^a^
Yes	16 (45.7)	10 (47.6)	
Liver metastasis			
No	24 (68.6)	15 (71.4)	0.822 ^a^
Yes	11 (31.4)	6 (28.6)	
Treatment response			
Partial	14 (40)	1 (4.8)	<0.001 ^b^
Complete	0 (0)	4 (19)	
Stabil	13 (37.1)	5 (23.8)	
Progression	8 (22.9)	11 (52.4)	

^a^: Pearson Chi Square test, ^b^: Fisher’s Exact test, ^c^: Mann Whitney U test, *p* < 0.05.

## Data Availability

The data that support the findings of this study are not openly available due to reasons of sensitivity and are available from the corresponding author upon reasonable request.
